# Use of anthropometric indicators in screening for undiagnosed vertebral fractures: A cross-sectional analysis of the Fukui Osteoporosis Cohort (FOC) study

**DOI:** 10.1186/1471-2474-9-157

**Published:** 2008-11-26

**Authors:** Kiyoko Abe, Junko Tamaki, Eiko Kadowaki, Yuho Sato, Akemi Morita, Misa Komatsu, Sayaka Takeuchi, Etsuko Kajita, Masayuki Iki

**Affiliations:** 1 Department of Public Health, Kinki University School of Medicine, Osaka-sayama, Japan; 2 Department of Domestic Sciences, Jin-Ai Women's College, Fukui, Japan; 3 Nutritional Epidemiology Program, National Institute of Health and Nutrition, Tokyo, Japan; 4 Seijyuji Nursing College, Mie, Japan; 5 Kosai City Office, Shizuoka, Japan; 6 Department of Public Health and Home Nursing, Nagoya University School of Health Science, Nagoya, Japan

## Abstract

**Background:**

Vertebral fractures are the most common type of osteoporotic fracture. Although often asymptomatic, each vertebral fracture increases the risk of additional fractures. Development of a safe and simple screening method is necessary to identify individuals with asymptomatic vertebral fractures.

**Methods:**

Lateral imaging of the spine by single energy X-ray absorptiometry and vertebral morphometry were conducted in 116 Japanese women (mean age: 69.9 ± 9.3 yr). Vertebral deformities were diagnosed by the McCloskey-Kanis criteria and were used as a proxy for vertebral fractures. We evaluated whether anthropometric parameters including arm span-height difference (AHD), wall-occiput distance (WOD), and rib-pelvis distance (RPD) were related to vertebral deformities. Positive findings were defined for AHD as ≥ 4.0 cm, for WOD as ≥ 5 mm, and for RPD as ≤ two fingerbreadths. Receiver operating characteristics curves analysis was performed, and cut-off values were determined to give maximum difference between sensitivity and false-positive rate. Expected probabilities for vertebral deformities were calculated using logistic regression analysis.

**Results:**

The mean AHD for those participants with and without vertebral deformities were 7.0 ± 4.1 cm and 4.2 ± 4.2 cm (p < 0.01), respectively. Sensitivity and specificity for use of AHD-positive, WOD-positive and RPD-positive values in predicting vertebral deformities were 0.85 (95% CI: 0.69, 1.01) and 0.52 (95% CI: 0.42, 0.62); 0.70 (95% CI: 0.50, 0.90) and 0.67 (95% CI: 0.57, 0.76); and 0.67 (95% CI: 0.47, 0.87) and 0.59 (95% CI: 0.50, 0.69), respectively. The sensitivity, specificity, and likelihood ratio for a positive result (LR) for use of combined AHD-positive and WOD-positive values were 0.65 (95% CI: 0.44, 0.86), 0.81 (95% CI: 0.73, 0.89), and 3.47 (95% CI: 3.01, 3.99), respectively. The expected probability of vertebral deformities (P) was obtained by the equation; P = 1-(exp [-1.327-0.040 × body weight +1.332 × WOD-positive + 1.623 × AHD-positive])^-1^. The sensitivity, specificity and LR for use of a 0.306 cut-off value for probability of vertebral fractures were 0.65 (95% CI: 0.44, 0.86), 0.87 (95% CI: 0.80, 0.93), and 4.82 (95% CI: 4.00, 5.77), respectively.

**Conclusion:**

Both WOD and AHD effectively predicted vertebral deformities. This screening method could be used in a strategy to prevent additional vertebral fractures, even when X-ray technology is not available.

## Background

Osteoporosis is defined as the loss of bone mass and deterioration of bone quality. Progression of this condition results in fragile bones and an increased risk of fractures [1]. The goal of osteoporosis prevention and treatment, therefore, is to prevent fractures. Because epidemiologic research has shown that low bone density is an important risk factor for fracture [2], osteoporosis screening in Japan is based on bone densitometry. Low bone density is not the only risk factor for osteoporosis, however, and this parameter alone has not proven to be sufficient for predicting fracture risk [3,4]. Recently, a research group from the World Health Organization (WHO) proposed a more accurate algorithm for assessing fracture risk, which includes clinical risk factors such as low body mass index (BMI) and a history of fractures in addition to bone mineral density (BMD) [5,6].

A history of fractures is one of the major risk factors for subsequent fractures, and an existing vertebral fracture increases the risk for new vertebral fractures by five-fold [7]. In comparison, BMD would have to decrease by three standard deviations to achieve an equivalent increase in risk [8]. While measuring BMD is important for predicting the risk of vertebral fractures, detection of undiagnosed fractures may play an even more critical role.

Vertebral fractures are the most common type of osteoporotic fracture [9], and are often asymptomatic [10]. Only 30% of individuals who have suffered vertebral fractures, in fact, visit a health care provider with symptomatic complaints [10]. It is therefore necessary to establish a screening method to detect existing vertebral fractures in asymptomatic individuals.

Researchers have attempted to develop simple screening methods for detecting undiagnosed vertebral fractures without the use of spinal X-ray imaging [11-17]. Most of these methods focus on detecting deformities in spinal alignment caused by vertebral fractures through anthropometry or manual examinations [11,14,15]. In particular, reduced body height has been investigated as a possible predictor of undiagnosed vertebral fractures [12,16,17]. However, it is often difficult to determine accurately longitudinal changes in body height. Some have proposed to use arm span (AS) as a proxy for the peak height of an individual, as this parameter tends to remain constant with advancing age [13,18]. The difference between AS and current body height (arm span-height difference: AHD) could therefore be used to detect undiagnosed vertebral fractures [11-13].

While a recent review by Green et al. [12] concluded that AHD did not predict undiagnosed fractures, these authors did identify a positive correlation between vertebral fractures and a positive wall-occiput test. A positive test result is defined as the inability to touch the wall with the back of the head when standing with back and heels against the wall. Although the back of the head normally touches the wall while maintaining a horizontal orbito-mental line, kyphotic spinal curvatures prevent the back of the head from easily reaching the wall. Green et al. [12] reported that the distance between the back of the head and the wall (wall-occiput distance: WOD) could be used to screen for undiagnosed vertebral fractures. A third anthropometric parameter of interest is the distance between the eleventh rib and iliac crest (rib-pelvis distance: RPD), measured manually. A reduction in RPD may be a sign of vertebral fracture, because the kyphotic spinal curvature induced by vertebral fractures causes the eleventh rib to approximate the iliac crest more closely [12,14].

While each of these screening techniques has been examined in Caucasian populations [12,14,17], little research to date has focused on individuals of Asian descent. Because Japanese individuals are generally shorter than Caucasians, standard cut-off values for reductions in height or AHD are not generally applicable to Japanese populations. However, it may be possible to measure WOD and RPD values more accurately in Japanese individuals, who generally have lower BMI and less probability of obesity [19,20] than Caucasian individuals.

Interestingly, each anthropometric parameter has been evaluated separately [20,27], but no attempt has yet been made to use them concomitantly in a screening evaluation for vertebral fractures. The authors of this study, therefore, evaluated the efficacy of using AHD, WOD, and RPD separately and in combination to screen for undiagnosed vertebral fractures in middle-aged and elderly Japanese women.

## Methods

### Participants and setting

Participants in this study were drawn from a follow-up survey conducted in 2005 as a part of the Fukui Osteoporosis Cohort Study [21-23]. The cohort study was initiated in 1990 and examined women between the ages of 35 and 72 years living in the Fukui Prefecture of Japan. Of the 177 women examined at baseline after excluding subjects with deformities of the lumbar spine, histories of oophorectomy, or lack of complete menopause histories, 116 who participated in the follow-up study in 2005 were used for analysis.

### Spinal vertebrae morphometry and diagnosis of vertebral deformities

The thoracolumbar vertebrae of each participant were imaged by single energy X-ray absorptiometry (QDR4500A (vehicle-mounted), Hologic Inc., USA). Bone morphometric software (QDR4500A Lateral Image Analyze, Hologic Inc., USA) was used to measure the anterior edge height, central height, and the posterior edge height of each vertebra from the fourth thoracic vertebra to the fourth lumbar vertebra. The McCloskey-Kanis criteria [24] were used to diagnose vertebral deformities, which were used as a proxy for vertebral fractures in the present study. The measurements of vertebral body height were conducted by a single trained investigator. One physician checked images and point-placements when an unusual measurement was observed, and vertebral heights were measured again.

### Bone Mineral Density Measurement

BMD was measured at the second, third, and fourth lumbar vertebrae using dual energy X-ray absorptiometry (QDR4500A (vehicle-mounted), Hologic Inc.,USA). The coefficient of variation (CV) for this measurement was 1.2% [25]. The BMD Z-score was calculated with age-stratified mean and standard deviation values for lumbar BMD, as defined in the JPOS Cohort Study [25].

### Anthropometric Measurements

Body height (cm) and weight (kg) were measured using an automatic scale (Takei Kagaku, model TK-11868h) with unit increments of 0.1 cm and 0.1 kg, respectively. The CV was 0.1% for body height and 0.9% for weight; CV values were calculated by measuring three subjects with a total of 28 times.

#### 1. Arm span-height difference

Two trained public health nurses performed all AS measurements throughout the survey. To obtain AS measurements, each participant was asked to face a wall with her arms at a 90° angle from the trunk and her palms against the wall. The distance between the tips of her middle fingers in unit increments of 0.1 cm was then measured. The CV for AS, determined by measuring two subjects a total of 28 times, was 0.4%. AHD was defined as the difference between body height and AS.

#### 2. Wall-occiput distance

Two trained public health nurses performed all measurements. Each participant was asked to stand straight against a wall with her heels, buttocks, and back touching the wall, and with her head position set to maintain a horizontal orbito-mental line. The horizontal distance between the wall and the back of the head was measured in increments of 5 mm. Values of at least 5 mm were considered WOD-positive.

#### 3. Rib-pelvis distance

Two medical doctors performed these measurements for all participants. The rib-pelvis distance was defined as the vertical distance between the inferior margin of the eleventh rib and the superior surface of the iliac crest along the midaxillary line, and was measured by the physician, who stood behind the participant. RPD was measured in units of 0.5 fingerbreadths, with two or less fingerbreadths constituting a positive test [14].

### Statistical Analysis

We performed a cross-sectional analysis of the association between existing vertebral deformities and the anthropometric indicators obtained in the 2005 survey. Participants were age-stratified into groups at 10-year intervals, and inter-group comparisons were carried out for age, body weight, body height, AS, AHD, BMD, and BMD Z-score using a one-way analysis of variance. A t-test was also used to compare age and body weight between the group of participants with positive test outcomes and the group with negative test outcomes, for each anthropometric test. A chi-squared test was used to compare prevalence rates for vertebral deformities between the groups. 

In order to derive the most appropriate AHD cut-off value for use in detecting existing vertebral deformities, receiver operating characteristic (ROC) curves were constructed. The value that maximized the difference between sensitivity and the false positive rate (the Youden index [26]) was selected as the cut-off value.

For each anthropometric indicator, the following statistical measures were calculated, along with 95% confidence intervals (95% CI) for each measure: sensitivity, specificity, and likelihood ratio for a positive result (LR) of vertebral deformities. A logistic regression analysis for vertebral fractures was conducted using body weight and the anthropometric indicators to estimate the expected probability (P) for existing vertebral deformities. Dummy variables for the anthropometric indicators were employed for the analysis (1 = positive results; 0 = negative results). To evaluate the goodness-of-fit of the regression equations to the data, Akaike information criterion (AIC) was calculated with the following equation,

AIC = -2(logM - k)

for which M was the maximum likelihood of a model and k was the number of independently adjusted parameters in the model [27]. When a difference in AIC between two models is more than 1, the model with the smaller AIC is considered to be a significantly better fit for the data [28]. ROC analysis of the expected probability calculated with the best fitted model was again performed to identify appropriate cut-off values of P for detecting vertebral deformities. Finally, sensitivity, specificity, and LR were calculated for each combination of anthropometric measures. Statistical analyses were performed with SPSS (version 14.0J; SPSS, Tokyo, Japan).

The study protocol in compliance with the Helsinki Declaration was approved by the Ethics Committee of the Kinki University School of Medicine. Written informed consent regarding all study procedures was obtained in advance from each participant in the study.

## Results

### Basic characteristics of the participants

Basic characteristics of the participants by 10-year age groups are shown in Table 1. While body height was reduced with increasing age, no significant differences in AS were noted between age groups; AHD therefore increased with advancing age. The proportions of WOD-positive and RPD-positive test results were positively correlated with age. The mean AHD value and the proportions of WOD positive and RPD positive results for participants with vertebral deformities were greater than those participants without vertebral deformities (Table 2). The distributions of the anthropometric indicators with or without vertebral deformities by age group are displayed in Table 3. Twenty participants were diagnosed with vertebral deformities. Of these, deformities were found in the thoracic spine in 12 participants, in the lumbar spine in 12 participants, and in both regions in 4 participants. The total number of vertebral deformities was 36, which included 17 vertebrae at the thoracic spine and 19 vertebrae at the lumbar spine. Two deformities were classified as concave type, 8 as crush type, and 26 as wedge type. One participant only had a concave type vertebral deformity. The numbers of vertebrae with deformities was 2 in 4 participants, and 3 or more in 4 other participants.

**Table 1:** Anthropometric indicators and bone mineral density of participants in the FOC Study, 2005.

	Total (n = 116) mean ± SD	49 – 59 years (n = 22) mean ± SD	60 – 69 years (n = 26) mean ± SD	70 – 79 years (n = 52) mean ± SD	80 years – (n = 16) mean ± SD	P^1^
Age (year)	69.9 ± 9.3	54.8 ± 2.9	65.8 ± 2.4	74.2 ± 2.8	83.1 ± 2.1	
Weight (kg)	52.2 ± 8.4	54.3 ± 7.6	53.5 ± 10.6	51.4 ± 8.1	50.1 ± 5.9	0.332
Height (cm)	148.0 ± 6.4	152.1 ± 4.8	149.1 ± 6.9	146.6 ± 6.1	145.4 ± 6.3	0.001
AS (cm)	152.8 ± 6.7	153.1 ± 6.5	154.0 ± 7.3	152.5 ± 7.1	151.2 ± 4.1	0.586
AHD (cm)	4.7 ± 4.3	1.0 ± 3.5	4.9 ± 4.6	5.9 ± 3.5	5.8 ± 4.5	< 0.001
WOD positive^2 ^n (%)	46 (40%)	0	1 (4%)	32 (62%)	13 (81%)	< 0.001
RPD positive^3 ^n (%)	52 (45%)	2 (9%)	9 (35%)	32 (62%)	9 (56%)	< 0.001
Subjects with vertebral deformities n (%)	20 (17%)	1 (5%)	3 (12%)	12 (23%)	4 (25%)	0.172

	(n = 108)	(n = 22)	(n = 24)	(n = 47)	(n = 15)	
Lumbar spine BMD (g/cm^2^)	0.812 ± 0.145	0.919 ± 0.156	0.827 ± 0.135	0.775 ± 0.122	0.751 ± 0.134	< 0.001
Lumbar spine BMD Z-score	0.317 ± 1.074	0.219 ± 1.126	0.268 ± 1.028	0.268 ± 1.055	0.692 ± 1.159	0.546

FOC Study: Fukui Osteoporosis Cohort Study

AS: Arm span, AHD: Arm span-height difference, WOD: Wall-occiput distance, RPD: Rib-pelvis distance, BMD: Bone mineral density

^1^P-values were obtained by the analysis of variance (ANOVA) or chi-squared test to compare anthropometric indicators and bone mineral density across age groups.

^2^WOD positive: ≥ 5 mm

^3^RPD positive: ≤ 2 fingerbreadths

**Table 2:** Anthropometric indicators and bone mineral density of subjects with or without vertebral deformities.

	Subjects with vertebral deformities n = 20 (17%)	Subjects without vertebral deformities n = 96 (83%)	
	mean ± SD	mean ± SD	P-value^1^
Age (years)	73.6 ± 6.2	69.1 ± 9.7	0.012
Weight (kg)	50.4 ± 7.1	52.6 ± 8.6	0.298
Height (cm)	144.3 ± 6.5	148.8 ± 6.2	0.003
AS (cm)	151.3 ± 7.1	153.1 ± 6.6	0.281
AHD (cm)	7.0 ± 4.1	4.2 ± 4.2	0.007
WOD positive ^2 ^n (%)	14 (70%)	32 (33%)	0.002
RPD positive ^3 ^n (%)	13 (65%)	39 (41%)	0.046

	(n = 12)	(n = 96)	
Lumbar spine BMD (g/cm^2^)	0.756 ± 0.107	0.820 ± 0.148	0.152
Lumbar spine BMD Z-score	0.047 ± 0.903	0.351 ± 1.093	0.358

AS: Arm span, AHD: Arm span-height difference

WOD: Wall-occiput distance, RPD: Rib-pelvis distance, BMD: Bone mineral density

^1^P-value by t-test or chi-squared test

^2 ^WOD positive: ≥ 5 mm

^3 ^RPD positive: ≤ 2 fingerbreadths

**Table 3:** Anthropometric indicators and bone mineral density of subjects with or without vertebral deformities by age-groups.

	49–59 years (n = 22)		60 – 69 years (n = 26)		70 – 79 years (n = 52)		80 years – (n = 16)	
	Subjects with vertebral deformities (n = 1)	Subjects without vertebral deformities (n = 21)	Subjects with vertebral deformities (n = 3)	Subjects without vertebral deformities (n = 23)	Subjects with vertebral deformities (n = 12)	Subjects without vertebral deformities (n = 40)	Subjects with vertebral deformities (n = 4)	Subjects without vertebral deformities (n = 12)
	M	M ± SD	M ± SD	M ± SD	M ± SD	M ± SD	M ± SD	M ± SD
Age (year)	56	54.8 ± 3.0	68.3 ± 1.2	65.4 ± 2.3	73.6 ± 2.4	74.4 ± 2.9	81.8 ± 2.1	83.5 ± 2.0
Weight (kg)	66.2	53.7 ± 7.3	50.2 ± 9.7	53.9 ± 10.8	49.4 ± 5.0	52.0 ± 8.8	49.9 ± 8.9	50.2 ± 5.0
Height (cm)	149.2	155.2 ± 4.9	144.9 ± 7.1	149.7 ± 6.8	143.5 ± 7.1	147.5 ± 5.5	144.8 ± 5.8	145.6 ± 6.6
AS (cm)	148.8	153.3 ± 6.4	154.5 ± 6.5	154.0 ± 7.8	150.6 ± 8.4	153.0 ± 6.6	151.7 ± 4.9	151.1 ± 4.2
AHD (cm)	-0.4	1.10 ± 3.5	9.6 ± 4.5	4.3 ± 4.3	7.1 ± 3.8	5.5 ± 3.4	6.9 ± 3.7	5.5 ± 4.9
WOD positive ^1 ^n (%)	0	0	1 (33%)	0	9 (75%)	23 (58%)	4 (100%)	9 (75%)
RPD positive ^2 ^n (%)	0	2 (9%)	3 (100%)	6 (26%)	8 (67%)	24 (60%)	2 (50%)	7 (59%)

	(n = 1)	(n = 21)	(n = 1)	(n = 23)	(n = 7)	(n = 40)	(n = 3)	(n = 12)
Lumbar spine BMD (g/cm^2^)	0.910	0.920 ± 0.160	0.840	0.826 ± 0.134	0.711 ± 0.091	0.786 ± 0.124	0.780 ± 0.121	0.743 ± 0.141
Lumbar spine BMD Z-score	0.271	0.217 ± 1.154	0.541	0.256 ± 1.049	0.393 ± 0.784	0.384 ± 1.061	0.835 ± 0.940	0.656 ± 1.241

M: means, AS: Arm span, AHD: Arm span-height difference, WOD: Wall-occiput distance, RPD: Rib-pelvis distance, BMD: Bone mineral density

^1 ^WOD positive: ≥ 5 mm

^2 ^RPD positive: ≤ 2 fingerbreadths

### The sensitivity, specificity, and LR findings for each anthropometric indicator

**Figure 1:**
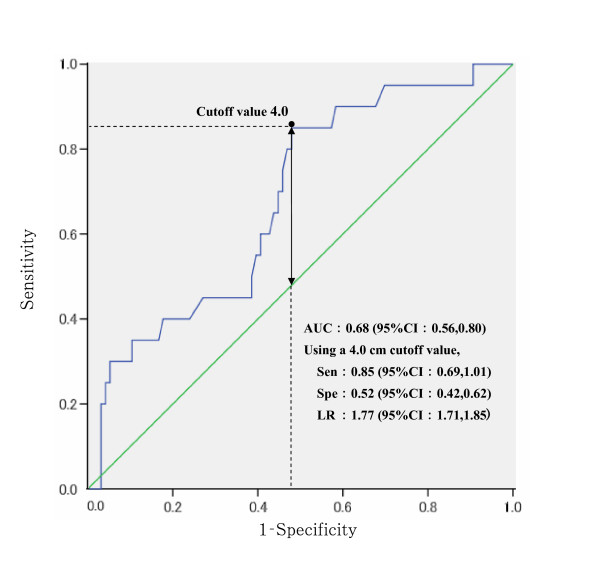
**ROC analysis of AHD for detection of existing vertebral deforimities**. AHD: Arm span-height difference, AUC: Area under the curve. 95%CI: 95% confidence interval, Sen: Sensitivity, Spe: Specificity, LR: Likelihood ratio for a positive result. Cutoff values were determined from the Youden index [26]. Arrow indicates the Youden index [26].

The ROC analysis found that an AHD cut-off value of 4.0 cm was most appropriate for detection of existing vertebral deformities, with an LR of 1.77 (95% CI: 1.71, 1.85) (Figure 1). This value was used for subsequent analysis.

Table 4 displays the sensitivity, specificity, and LR findings for each anthropometric indicator. Neither the sensitivity nor specificity of RPD was significant. Significantly higher rates of vertebral deformities were found in the groups with positive WOD and AHD results (Table 5), but in each case, the group with positive results was significantly older than the group with negative results.

**Table 4:** Sensitivity, specificity, and likelihood ratio for anthropometric indicators used to detect vertebral deformities.

	Sensitivity	95% CI	Specificity	95% CI	LR	95% CI
AHD positive^1^	0.85	(0.69, 1.01)	0.52	(0.42, 0.62)	1.77	(1.71, 1.85)
WOD positive^2^	0.70	(0.50, 0.90)	0.67	(0.57, 0.76)	2.10	(1.93, 2.28)
RPD positive^3^	0.67	(0.47, 0.87)	0.59	(0.50, 0.69)	1.60	(1.47, 1.74)

LR: Likelihood ratio for a positive result, 95% CI: 95% confidence interval

AHD: Arm span-height difference, WOD: Wall-occiput distance

RPD: Rib-pelvis distance

^1^AHD positive: ≥ 4.0 cm

^2^WOD positive: ≥ 5 mm

^3^RPD positive: ≤ 2 fingerbreadths

**Table 5:** Age, weight and prevalence of vertebral deformities by AHD and WOD test results.

		Age (year)	Weight (kg)	Number of subjects with vertebral deformities (%)
		mean ± SD	mean ± SD	
AHD^1^	Positive (n = 63)	72.4 ± 7.6	52.4 ± 8.8	17 (27%)
	Negative (n = 53)	66.8 ± 10.3	52.0 ± 8.0	3 (6%)
	P^3^	0.001	0.755	0.003

WOD^2^	Positive (n = 46)	76.8 ± 4.7	52.1 ± 7.2	14 (30%)
	Negative (n = 70)	65.3 ± 8.7	52.3 ± 9.2	6 (9%)

	P^3^	< 0.001	0.906	0.002

AHD: Arm span-height difference, WOD: Wall-occiput distance

^1^AHD positive: ≥ 4.0 cm, AHD negative: < 4.0 cm.

^2^WOD positive: ≥ 5 mm, WOD negative: < 5 mm.

^3^P-value by t-test or chi-squared test.

### Comparison of the models for vertebral deformities

**Figure 2:**
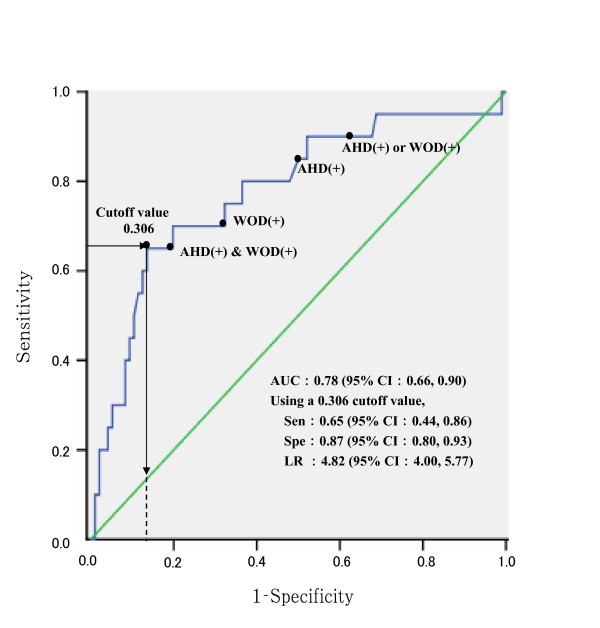
**ROC analysis of the expected probability for detection of existing vertebral deformities**. WOD: Wall-occiput distance, AHD: Arm span-height difference, Sen: Sensitivity, Spe: Specificity, LR: Likelihood ratio for a positive result, AUC: Area under the curve, 95% CI: 95% confidence interval, WOD ( + ): values of 5 mm or above, AHD ( + ): values of 4.0 cm or above. Arrow indicates the Youden index [26]. The solid line shows the ROC curve of vertebral fractures by the expected probability of existing vertebral deformities. The expected probability (P) of existing vertebral fractures was calculated using the following formula: P = 1-(exp[-1.327–0.040 × body weight+1.332 × WOD-positive+1.623 × AHD-positive])^-1^, where positive results for WOD or AHD were assigned a value of 1, and negative results were assigned a value of 0. The cutoff value was determined from the Youden index [26]. The black circle (●) in the figure identifies sensitivity and specificity values for WOD and AHD, both independently and in combination.

Logistic regression models for vertebral deformities were constructed using WOD and AHD results using various combinations of age and body weight as independent variables. Both anthropometric variables were significant in all models. In the simplest model comprising AHD and WOD only (Model 1), the regression coefficients were 1.329 and 1.614, respectively, and the AIC was calculated to be 96.28. Similarly, in a logistic regression model using AHD, WOD and weight (Model 2), AIC was 96.96. The model with AHD, WOD, age and weight (Model 3) showed AIC of 98.75. According to AIC values, Model 1 and Model 2 fit the data significantly better than did Model 3, but no significant difference in goodness-of-fit was observed between Models 1 and 2. The following formula was derived from Model 2 to estimate the expected probability of vertebral deformities (P):

P = 1-(exp [-1.327-0.040 × body weight +1.332 × WOD-positive + 1.623 × AHD-positive])^-1^, where we assigned a value 1 for positive results of WOD and AHD and a value 0 for negative results. Using the Youden index, the cut-off value for P was set at 0.306 to predict existing vertebral deformities most accurately, as found in the ROC analysis (Figure 2).

Because body weight did not have a statistically significant regression coefficient in Model 2, it could be removed from the equation, which left only two binomial variables in the model. This model could be broken down into the combination of WOD and AHD test results. Table 6 displays the sensitivity, specificity, and LR for each combination of results for WOD and AHD indicators. The LR for existing vertebral deformities was highest with positive results for both WOD and AHD; the LR value in this scenario was nearly equivalent to the LR from the full logistical regression diagnostic model for which body weight was included (Figure 2).

**Table 6:** Sensitivity, specificity, and likelihood ratio for use of combined anthropometric indicators in detecting vertebral deformities.

Criteria for positive results	No. of subjects with:				Sensitivity (95% CI)	Specificity (95% CI)	LR (95% CI)
	TP	FN	TN	FP			
AHD positive^1^	17	3	50	46	0.85 (0.69, 1.01)	0.52 (0.42, 0.62)	1.77 (1.71, 1.85)
WOD positive^2^	14	6	64	32	0.70 (0.50, 0.90)	0.67 (0.57, 0.76)	2.10 (1.93, 2.28)
AHD positive and WOD positive	13	7	78	18	0.65 (0.44, 0.86)	0.81 (0.73, 0.89)	3.47 (3.01, 3.99)
AHD positive or WOD positive	18	2	36	60	0.90 (0.77, 1.03)	0.38 (0.28, 0.47)	1.44 (1.41, 1.47)

TP: True positive, FN: False negative, TN: True negative, FP: False positive

LR: Likelihood ratio for a positive result

95% CI: 95% confidence interval, AHD: Arm span-height difference

WOD: Wall-occiput distance, AHD positive: ≥ 4.0 cm, WOD positive: ≥ 5 mm

^1^AHD positive: AHD positive, and WOD positive or negative.

^2^WOD positive: WOD positive, and AHD positive or negative.

## Discussion

The results of this study indicate that WOD and AHD, either independently or in combination, can be used as an effective screening method to detect undiagnosed vertebral deformities. In addition, using three parameters of WOD, AHD, and body weight was found to barely increase the screening efficacy. Our study is the first to indicate that it is possible to screen middle-aged and elderly women for undiagnosed vertebral deformities by combining these anthropometric indicators.

Growing evidence surrounding the adverse outcomes associated with vertebral fractures [29-31] suggests that such screening programs are worthwhile. Increasing numbers of fractured vertebrae have been shown to increase back pain and reduce both quality of life [29] and the ability to perform activities of daily living [30]. In addition, the life expectancy of individuals with vertebral fractures is reportedly lower than that of individuals who have suffered non-traumatic radial or humeral fractures [31].

Previous studies have found AHD to be an insufficient predictor of undiagnosed vertebral fractures [11,13]. Versluis et al. [11] found no significant difference in AHD values between individuals with and without vertebral fractures when they used the Eastell method [32] as a diagnostic gold standard for vertebral fractures. Similarly, a study by Wang et al. [13] compared AHD values between healthy controls (average age: 58 years) and patients with vertebral fractures resulting in a vertebral height reduction greater than 20% (average age: 71 years). No differences in AHD values were identified between the two groups.

The negative results of these studies [11,13] may be due to the methodology employed. The criteria for each study, which were an Eastell Grade I vertebral fracture and a vertebral height reduction greater than 20%, respectively, selected for subjects with only minor morphometric vertebral changes. This choice of subject groups may explain the lack of statistically significant reductions in body height. In support of this theory, Versluis et al. [11] did find significant differences in AHD values between patients with Eastell Grade II fractures and patients without fractures or those with Grade I fractures. In both studies, AS was measured in participants who stood with their backs against a wall [11,13], a method which may lead to inaccurate measurements in subjects with kyphosis. AS measurements in these studies, therefore, cannot be considered to be proxy measurements for peak body height. In our study, we measured AS with participants facing a wall, in order to maximize the accuracy of the measurements.

An additional explanation for the effectiveness of AHD in the present study may be that Japanese individuals have a higher risk for severe reduction in vertebral height and multiple vertebral fractures than Caucasian individuals [33]. The degree to which body height is reduced in subjects with vertebral fractures may therefore be greater in Japanese than in Caucasians. Moreover, the distribution of vertebral fractures in Japanese individuals is weighted more heavily in the lumbar vertebrae, as compared to Caucasians [33]. Each of these features may contribute to greater reductions in body height among Japanese with vertebral fractures [34]. We therefore conclude that AHD is an effective screening measure for undiagnosed vertebral deformities in Japanese women. We also expect it to be a valid measure for other Asian women with similar bone structures and physiques.

Green et al. [12] recently reviewed a study by Siminoski et al., and reported that WOD was an effective screening indicator for vertebral fractures. According to the review, Siminoski et al. found screening with WOD to be 60% sensitive and 87% specific. The authors also calculated the LR to be 4.6 (95% CI: 2.9, 7.3) for WOD screening when WOD values greater than zero were defined as positive in 213 female Canadian outpatients. In contrast, Balzini et al. [35] found no correlation between WOD and vertebral fractures. However, their particular study had an insufficient sample size of only 60 participants. It is also possible that the criteria [36] used by Balzini et al. [35] to diagnose vertebral fractures differ from those used in this study. The results of our study suggest that the use of WOD measurements is a simple and effective screening method for undiagnosed vertebral deformities, and could be used in both European and Asian populations.

RPD has been reported to be effective in screening for vertebral fractures [14], and that RPD values are lower in subjects with lumbar vertebral fractures than in subjects with thoracic vertebral fractures [14]. In our study, however, we found no correlation between RPD and vertebral deformities. One possible explanation for this discrepancy may be due to false positives produced by the McCloskey-Kanis criteria [24] in the present study. The other explanation may be that the use of fingerbreadths as a measurement technique was inexact and difficult to reproduce.

There are several limitations in our study. The most important one may be that we used vertebral morphometry with the McCloskey-Kanis criteria to identify the cases with vertebral fractures. As such, our diagnostic results may have included a number of false positives and negatives. The prevalence rate of false positives produced by this method was reported as 16% [24]. Supplementing this method with visual semi-quantitative methods including the Genant's method, as recommended by the 2007 ISCD Official Positions, may have increased our accuracy for vertebral fracture detection [37]. Jiang et al. [38] developed a modified visual diagnostic method which requires the depression of endplate for the diagnosis of osteoporotic vertebral fracture. Use of the method by Jiang et al. [38] may have reduced the false positive rates in the identification of cases with vertebral fractures in our study.

We also used X-ray absorptiometry instead of conventional radiographs, which may have resulted in missing some cases with vertebral fractures. Rea et al. [39] indicated relatively good agreement (κ = 0.75 per subject) in the diagnosis of vertebral fractures in postmenopausal women formed by McCloskey-Kanis criteria using X-ray absorptiometry versus conventional radiographs. However, X-ray absorptiometry tends to miss concave type, grade 1 fractures according to Genant's semi-quantitative grading or endplate fractures. Ross et al. reported that the proportion of concave type was lower in Japanese women than in Caucasian women [33], suggesting that the underestimation of vertebral fractures due to concave type would be smaller in magnitude in Japanese, as observed in the present study. However, it is important to realize that sensitivity values of the anthropometric method for vertebral fracture detection including mild types would be lower than values presented in this paper. This problem associated with X-ray absorptiometry, however, may not affect the efficacy of the anthropometric method in screening women with vertebral fractures because it is not expected to screen people with mild vertebral fractures which do not cause apparent spinal deformities. Therefore, the use of X-ray absorptiometry to measure vertebral body height was appropriate here, given the objectives of the current study. 

Another limitation of this study was the relatively small number of subjects. In particular, this made it difficult to analyze the association between the anthropometric indicators such as single versus multiple deformities, thoracic versus lumbar spine deformities, or with different types of vertebral fractures.

In terms of reproducibility, we did not determine the CV for vertebral body height measurements, though the McCloskey-Kanis criteria have been shown to have high test-retest and inter-observer reliability [40]. The CV for WOD was not measured either. It has been reported, however, that the correlation coefficients for repeated WOD measurements across six-month intervals in ankylosing spondylitis patients ranged between 0.94 and 0.96 [41], suggesting that our WOD measurements also would be relatively reliable. Additionally, we measured the CV for AS measurements in middle-aged women but not in elderly women. Shahar et al. [42] found that the CV for AS in women aged 60 to 86 years was quite similar to that in women aged 30 to 49 years. We therefore surmise that our AS measurements were reliable and valid.

## Conclusion

Obtaining WOD and AHD measurements during annual medical exams or osteoporosis screenings is an effective method of screening middle-aged and elderly women for undiagnosed vertebral fractures. This method is expected to contribute to both early diagnosis and prevention of additional fractures. In clinical situations where X-ray equipment is unavailable, we propose the following screening algorithm:

1) Screen using either WOD or AHD as independent indicators;

2) Use both indicators when clinical suspicion for undiagnosed vertebral fracture is high;

3) In situations where body weight data are available, calculate the probability of vertebral fractures with the formula shown in Figure 2.

Because this method can be used even in locations where X-ray equipment is not available, public health nurses could perform screenings for vertebral fractures during annual physical exams or health education classes. Widespread use of this screening method may lead to earlier diagnoses and increased awareness about osteoporosis and vertebral fractures. Because this method is expected to be applicable to non-Japanese women with similar bone structure and physique, use of anthropometric indicators can help prevent vertebral fractures in developing countries where X-ray equipment is not always available, and where osteoporosis will become an increasingly important issue.

## Competing interests

The authors declare that they have no competing interests.

## Authors' contributions

KA contributed substantially in the conduction of the study, performed the statistical analyses, and drafted and revised the manuscript. JT contributed substantially to the analyses and interpretation of the data, and contributed to the draft and revision of the manuscript. EK and YS assessed the vertebral deformities. AM, MK, and ST contributed substantially to conducting the study. EK contributed to the conception, design, and the conducting of the entire FOC study. MI is the principal investigator of the FOC study, and conceived, conducted the surveys, interpreted data, and gave final approval of the final version of the manuscript.

## Pre-publication history

The pre-publication history for this paper can be accessed here:


